# A novel mutation in the human RAX gene in patients with microphthalmia and coloboma: A case report and literature review

**DOI:** 10.1097/MD.0000000000048361

**Published:** 2026-04-24

**Authors:** Zhangyi Li, Shurui Ke, Wenjuan Wan, Ji Sun, Ke Hu, Can Li

**Affiliations:** a Department of Ophthalmology, Chongqing Emergency Medical Center, Chongqing University Center Hospital, The Fourth People’s Hospital of Chongqing, Chongqing, China; b Department of Ophthalmology, The First Affiliated Hospital of Chongqing Medical University; Chongqing Key Lab of Ophthalmology; Chongqing Eye Institute, Chongqing, China.

**Keywords:** coloboma, microphthalmia, mutation, RAX

## Abstract

**Rationale:**

Microphthalmia, anophthalmia, and coloboma (MAC) form a spectrum of severe developmental eye anomalies. Reports of human MAC associated with RAX mutations remain limited, exhibiting substantial phenotypic heterogeneity. Clinically managing these ocular anomalies or investigating their genetic mechanisms continues to present significant challenges.

**Patient concerns:**

A 28-year-old female and her 2 sisters L and Q presented with lifelong poor vision, ocular examination revealed similar phenotype among them, including narrow palpebral fissures, bilateral ptosis with epicanthus and esotropia, small cornea, iris coloboma and microspherophakia with uneven lens opacity, in addition to irreversible low vision.

**Diagnoses:**

Based on ocular examination, the proband was diagonosised with microphthalmia and coloboma, and her 2 sisters were diagonosised with coloboma. WES detected a novel RAX mutation in the 3 affected siblings.

**Interventions:**

Cataract extraction surgeries combined with complicate scleral fixation of intraocular lens were performed bilaterally in the proband and unilaterally in the right eyes of patient L and Q.

**Outcomes:**

The proband demonstrated improved bilateral visual acuity postoperatively, reaching 20/125 and 20/100. Patient Q’s right eye achieved 20/200 visual acuity, while Patient L’s right eye showed no change. The corrected intraocular pressure in Patient L’s right eye maintained below 21 mm Hg without requiring antiglaucoma medication.

**Lessons:**

The clinical management of MAC remains challenging. Identifying pathogenic genes could enable curative approaches for this condition. In pediatric patients with visual impairment, prompt intervention targeting ocular and systemic complications proves essential for optimizing visual outcomes.

## 1. Introduction

Microphthalmia describes eyes with axial lengths measuring at least 2 standard deviations below the age-adjusted mean, typically presenting as <21 mm in adults and <17 mm in newborns. Coloboma arises from incomplete embryonic optic fissure closure, causing tissue defects in the iris, retina, choroid, or sclera. Anophthalmia, the most severe ocular malformation, involves complete absence of intraorbital eye tissue while preserving adnexal structures like eyelids and lacrimal apparatus. These conditions – microphthalmia, anophthalmia, and coloboma (MAC) – form a spectrum of developmental eye anomalies with incidence rates of (0.4–3.3)/1,00,000 live births for anophthalmia, (10.0–14.3)/1,00,000 for microphthalmia, and 1/5000 for coloboma.^[1,2]^ Both genetic and environmental factors contribute to congenital ocular malformations. Genetic factors dominate the etiology, though these explain only 21% to 61% of clinical cases.^[3]^

Monogenic factor analysis has identified over 100 genes involved in ocular teratogenesis. Of all these genes, the retina and anterior neural fold homeobox (RAX) gene (OMIM: 601881), a paired homeobox gene with conserved sequence across vertebrate species, encodes a transcription factor essential for vertebrate eye and forebrain development.^[4]^ Germline and conditional Rax knockout models have been identified of anophthalmia alongside craniofacial, neurological abnormalities and perinatal lethality. Human cases of ocular malformations caused by RAX mutations have been documented, though clinical reports remain scarce. Mutations in the human RAX gene are associated with unilateral or bilateral ocular deformities, which may manifest either as isolated conditions or as prominent ocular defects within multisystem syndromes, demonstrating considerable phenotypic heterogeneity. In this study, we came across a consanguineous Chinese family in which the proband and her 2 sisters exhibited rare and similar ocular defects. To investigate the genetic basis, we performed whole-exome sequencing (WES) and identified a novel RAX gene mutation. Our work aims to expand the genotype-phenotype correlations of MAC spectrum.

## 2. Case report

A 28-year-old female presented to the clinic with lifelong poor vision but no prior ophthalmic diagnosis or treatment. Her visual acuity measured 20/333 in the right eye and 20/200 in the left, with no improvement after correction. The patient displayed narrow palpebral fissures, bilateral ptosis accompanied by epicanthus, esotropia with horizontal nystagmus, and an inability to fixate, which complicates standard ophthalmic examinations inlcuding fundus photography. Slit-lamp examination revealed a small cornea with abnormal thickness in both eyes and an iris coloboma positioned at 6 o’clock in the left eye. The lenses were opaque, exhibiting a tremulous globular appearance. Preoperative fundus examination was impossible due to cataract opacity, though postoperative fundus photography identified optic disk coloboma and defects in the choroid and retina, with only faint visibility. Both corneas measured 8 mm in diameter, and central corneal thicknesses were 729 μm in the right eye and 708 μm in the left eye. Axial lengths were 19.45 mm (the right eye) and 19.54 mm (the left eye).

**Figure 1.**
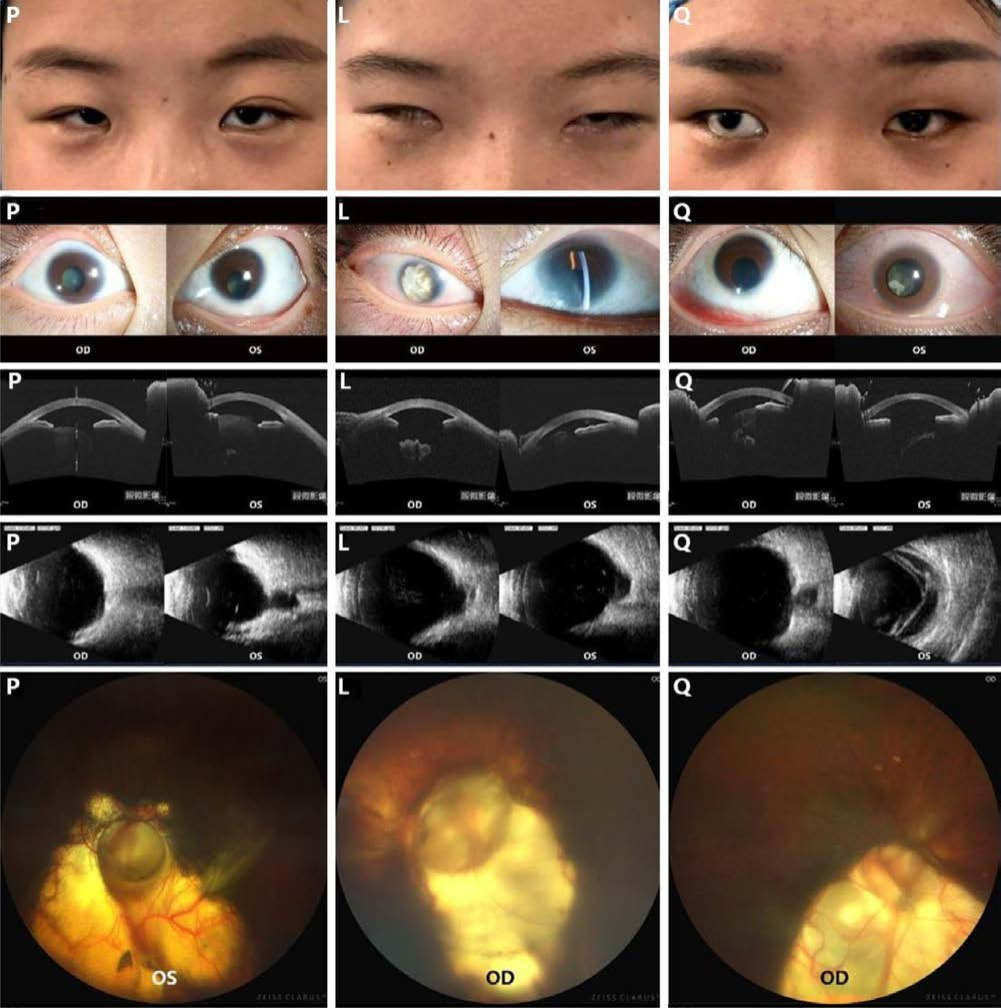
Clinical features of the 3 patients. Clinical pictures showed narrow palpebral fissures, bilateral ptosis accompanied by epicanthus and esotropia. Photographs of the anterior segments showed small cornea, iris coloboma and microspherophakia with uneven lens opacity. Anterior segment optical coherence tomography (AS-OCT) showed microspherophakia with uneven lens opacity, subluxation of lens and suspensory ligament relaxation. Ocular B-scan ultrasound indicated choroidal coloboma, optic disc coloboma and retinal detachment. Fundus photographs post cataract surgery showed optic disk coloboma and chorioretinal coloboma (P: the proband, L: patient L, Q: patient Q, OD: the right eye, OS: the left eye). AS-OCT = anterior segment optical coherence tomography.

**Figure 2.**
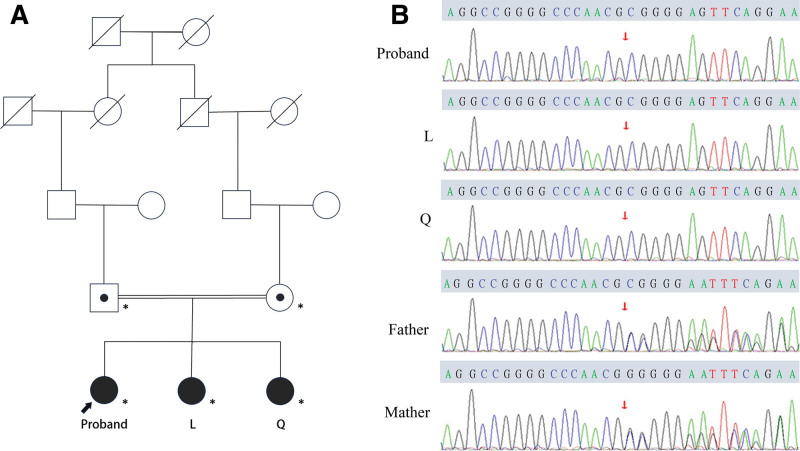
(A) Pedigrees of the consanguineous family. (B) Electropherograms showing a frameshift mutations in 3 patients (P: the proband, L: patient L, Q: patient Q, OD: the right eye, OS: the left eye).

The proband has 2 younger sisters, L and Q, who demonstrate similar ocular abnormalities, including congenital visual impairment and malformations (Fig. 1 and Table 1). In addition, all 3 siblings exhibit short stature, and the proband and Q have been diagnosed with small uteruses based on B-ultrasound measurements. Despite these findings, the patients show no deficits in intelligence, verbal expression, motor ability, or menstrual cycles. The family has a consanguineous background, yet physical and ocular examinations of the parents revealed no abnormalities (Fig. 2). WES detected a novel RAX mutation (c.863delC, p.P288Rfs*43) in the affected siblings and their parents (Fig. 2). This frameshift variant was classified as pathogenic based on ACMG criteria. All 3 patients were found to be homozygous for this mutation, while both parents were heterozygous carriers. Subsequently, cataract extraction surgeries combined with complicate scleral fixation of intraocular lens were performed bilaterally in the proband and unilaterally in the right eyes of patient L and Q. Both patients L and Q declined surgical intervention for their left eyes. Postoperative visual acuity in the right eye of patient L remained unchanged, whereas the proband exhibited bilateral improvement to 20/125 and 20/100. the right eye of patient Q similarly achieved a visual acuity of 20/200. the corrected intraocular pressure of patient L’s right eye maintained below 21 mm Hg postoperatively without antiglaucoma agents.

**Table 1:** Records of clinical data of the 3 patients.

	Proband	Patient L	Patient Q
General conditions			
Gender (F/M)	F	F	F
Age, yr	28	23	21
Height, cm	136	151	150
Weight, kg	38	61	62
Body mass index (BMI), kg/m^2^	20.54	26.75	27.56
Eye examinations			
Best corrected visual acuity (BCVA) in decimal record	OD: 20/333 OS: 20/250	OD: CF/10 cm OS: 20/800	OD: 20/250 OS: No light perception
Horizontal corneal diameter, mm	OD: 8.0 OS: 8.0	OD: 8.0 OS: 8.0	OD: 9.0 OS: 9.0
Central corneal thickness (CCT), µm	OD: 729 OS: 708	OD: 759 OS: 775	OD: 674 OS: 576
Corrected intraocular pressure (IOP), mm Hg	OD: 23.3 OS: 19.6	OD: 28.8 OS: 21.0	OD: 18.1 OS: 20.2
Length of axial, mm	OD: 19.45 OS: 19.54	OD: 24.38 OS: 23.78	OD: 22.54 OS: NA
Genital system			
Measurements of uterus in B ultrasound scan	The upper and lower diameters are about 62 mm The left and right diameters are about 38 mm The front and rear diameters are about 23 mm The thickness of the endometrium is about 3.5 mm	The upper and lower diameters are about 71 mm The left and right diameters are about 41 mm The front and rear diameters are about 31 mm The thickness of the endometrium is about 8 mm	The upper and lower diameters are about 62 mm The left and right diameters are about 38 mm The front and rear diameters are about 23 mm The thickness of endometrium is about 3.5 mm

BCVA = best corrected visual acuity, BMI = body mass index, CCT = central corneal thickness, IOP = intraocular pressure, NA = not available, OD = the right eye, OS = the left eye, RAX = the retina and anterior neural fold homeobox.

## 3. Discussion

In this study, we investigated a consanguineous Chinese family in which the proband and her 2 sisters exhibited rare but consistent ocular abnormalities. Subsequently, we successfully performed complex phacoemulsification surgeries combined with scleral-fixated intraocular lens implantation in all 3 patients. To uncover the genetic basis of these phenotypes, we conducted WES and variant analysis, identifying a novel mutation in the RAX gene as a potential causative factor. MAC are influenced by both genetic and environmental factors. While genetic alterations dominate, they explain only 21% to 61% of clinical cases. Previous studies on monogenic factors have identified over 100 genes implicated in ocular teratogenesis, including SOX2, OTX2, RAX, VSX2, PAX6, STRA6, and ALDH1A3, etc.^[3]^

Rax genes are present in all metazoan genomes, with the number of RAX paralogues varying among vertebrate species. Phylogenetic analysis classifies these genes into 2 groups: Rax1 and Rax2. It’s important to make it clear when comparing different studies. Humans possess 1 RAX1 gene (commonly referred to as RAX) and 1 RAX2 gene.^[5]^ The RAX1 gene is highly conserved across vertebrates. The RAX gene encodes methionine at position 10 in mammals like humans and rats, with this conserved site also identified in the equivalent position of Rx genes across fish, amphibians, and birds. The encoded protein functions as a transcription factor, essential for embryonic eye and forebrain development.^[6]^ In contrast, the RAX2 gene is exclusively expressed in the retina, where it supports lifelong retinal health.^[7]^ The RAX gene is located on chromosome 18 and consists of 3 exons and 2 introns. Its transcription begins in the anterior neural plate and subsequently appears in the eye field and ventral forebrain. As embryonic development proceeds, the gene is expressed in the retina, pituitary gland, hypothalamus, and pineal gland, with its expression in the adult retina significantly reduced.^[8]^ The human RAX protein comprises 346 amino acids and includes 4 evolutionarily conserved motifs: an N-terminal octapeptide region, a paired-class homeodomain, an Rx domain, and a C-terminal domain. Eye development is orchestrated by a tightly integrated and complex network of signaling factors, with RAX playing a pivotal role. Studies of Xenopus laevis have demonstrated that the synergistic effects of transcription factors OTX2 and SOX2 stimulate RAX gene expression, which, in turn, drives the production of downstream regulators Xhmgb3 and XOPTX2. These downstream factors collaboratively promote retinal cell proliferation. This signaling cascade is critical for proper eye development, and RAX dysfunction in this pathway can result in phenotypes such as MAC.^[1]^

Early studies initially suggested that heterozygous RAX mutations alone were insufficient to induce eye malformations, adhering to an autosomal recessive inheritance model. Voronina et al initially reported a case of compound RAX gene mutations (p.Q147X/p.R192Q), where the proband inherited each variant from his parents, who in turn acquired them from one of their own parents. Remarkably, the proband’s parents, grandmother, and maternal grandfather were asymptomatic carriers of the mutated RAX genes.^[2]^ As additional cases were reported, this hypothesis gained further support. Reports by Lequeux et al, Brachet et al, and the present case consistently identified healthy carriers with no ocular or systemic abnormalities tied to RAX mutations.^[1,8]^ Nonetheless, findings by London et al and Abouzeid et al challenged this conclusion, as they documented MAC patients harboring heterozygous RAX mutations.^[9,10]^ These patients exhibited distinct single-allelic mutations in the RAX gene, manifesting diverse clinical phenotypes of ocular developmental defects – ranging from retinal abnormalities to anophthalmia. Furthermore, some of these cases involved congenital nervous and motor system irregularities. These unrelated cases collectively suggest that even a single-allelic mutation in the RAX gene may disrupt ocular morphogenesis. Given the intricate processes underpinning eye development and the limited availability of corroborative data, the teratogenic potential of heterozygous RAX mutations remains an open question. Future research into the molecular mechanisms of the RAX gene will be essential in resolving these controversies and elucidating its role in eye formation.

According to current case reports, mutations in the human RAX gene can lead to ocular abnormalities, which may present unilaterally or bilaterally as isolated defects or as part of broader multisystem syndromes (Table 2). Biallelic mutations are commonly associated with severe ocular developmental disorders, such as anophthalmia and microphthalmia, often accompanied by nervous system and urogenital abnormalities. However, the 3 patients with homozygous RAX gene mutations described in this report exhibited milder clinical phenotypes, closely resembling the cases documented by Huang et al, featuring ocular defects as the main manifestation, except for the proband diagnosed with microphthalmia.^[12]^ The significant phenotypic and genotypic heterogeneity complicates the understanding of the relationship between RAX mutations and clinical outcomes, suggesting the potential involvement of complex and uncertain genetic regulatory mechanisms. Researchers applied different morpholino inhibitor concentrations to the RAX gene in Xenopus laevis, yielding a spectrum of phenotypes ranging from anophthalmia to microphthalmia, which indicates a dose–dependent correlation between RAX activity and ocular morphology.^[2]^ These findings suggest that reductions below the threshold of normal RAX activity likely exert a graded effect, with the most severe phenotypes associated with significant functional defects in the RAX protein. Furthermore, numerous studies have highlighted that the pathogenicity of RAX gene mutations is intricately linked to the location of the mutation, its type, and associated protein structure variations.

**Table 2:** Review of reported data of RAX mutations in humans.

Investigator	RAX mutation	Affected patients	Type of mutation	Ocular phenotype	Extraocular phenotype
Voronina et al^[2]^	Compound heterozygous p.Q147X/p.R192Q	1	Truncating/missense	Anophthalmia OD, sclerocornea with persistent fetal vasculature and retinal detachment OS	1. EEG showed abnormal slowing of background activity consistent with underlying cortical abnormality 2. Autistic
Lequeux et al^[8]^	Compound heterozygous p.Ser222-ArgfsX62/p.Tyr303X	1	Truncating/truncating	Bilateral anophthalmia	Optic nerves and chiasma were hypoplastic
London et al^[9]^	Heterozygous g.197G>C	1	Missense	Optic nerve coloboma OD	NA
Gonzalez-Rodriguez et al^[11]^	Heterozygous c.148a>c	1	NA	Microphthalmia OD	1. Septum pellucidum agenesis, cortical atrophy 2. Optic nerve atrophy OS
	Heterozygous c.328c>G	1	NA	Anophthalmia OD	Hydrocephalus, congenital hip dislocation
Abouzeid et al^[10]^	Homozygous c.54313A.G	2	Splicing	Bilateral anophthalmia	1. High arched palate 2. Hypoplastic orbit 3. Agenesis of the optic nerve, tracts and chiasm 4. The absence of frontal and sphenoidal sinuses
	Homozygous c.54313A.G	1	Splicing	Bilateral anophthalmia	1. Development delay, obesity, abnormal head circumference and a high arched palate 2. Hypoplastic orbit 3. Agenesis of the optic nerve, tracts and chiasm 4. The absence of sphenoidal sinuses 5. Corticosubcortical atrophy
Huang et al^[12]^	Homozygous c.113 T>C	1	Missense	Optic disk coloboma OD, iris and choroid coloboma OS	NA
Brachet et al^[1]^	Homozygous p.Pro89Argfs*114	1	Frameshift truncating	Bilateral anophthalmia	1. Bilateral cleft lip and palate 2. Micropenis 3. Panhypopituitarism and diabetes insipidus 4. No sella turcica and pituitary glands in MRI
Chassaing et al^[3]^	Compound heterozygous c.478T>C/c.563G>A	1	Missense/missense	Bilateral microphthalmia	Intellectual disability
	Homozygous c.560G>A	1	Missense	Bilateral anophthalmia	NA
	Compound heterozygous c.664delT/c.909C>G	1	NA	Bilateral anophthalmia	NA
	Compound heterozygous c.665C>A/c.(?_-30)_(*220_?)del	1	Nonsense/deletion	Bilateral anophthalmia	Pregnancy termination
Present report	Homozygous c.863delC	1	Frameshift	Bilateral microphthalmia	1. Short stature 2. Small uteruses
	Homozygous c.863delC	2	Frameshift	iris and chorioretinal coloboma	1. Short stature 2. Small uteruses

EEG = electroencephalogram, MRI = magnetic resonance imaging‌, NA = not available, OD = the right eye, OS = the left eye.

The surgeries for all 3 patients were completed successfully; however, postoperative visual function improvement remained limited. Despite this, both the proband and patient Q expressed pleasant surprise at their partial visual recovery, noting they had never seen the world with such clarity before. The modest improvement likely stems from partial defects in retinal and other critical ocular tissues, as well as visual developmental impairments caused by complications such as congenital cataract, uncontrolled intraocular pressure, retinal detachment, strabismus, and amblyopia. Consequently, enhanced focus should be directed toward comprehensive management strategies for MAC. For individuals at high risk of MAC, genetic counseling before conception and prenatal testing are crucial in reducing the likelihood of birthing severely malformed fetuses. For patients with severe microphthalmia or anophthalmia, exploring options such as ocular reconstructive surgery and artificial prosthetic transplantation can significantly enhance their physical appearance. Protecting the healthy eye should be a priority for patients with unilateral involvement.^[13]^ For visually impaired children, early interventions targeting complications like cataracts, glaucoma, refractive errors, strabismus, and amblyopia, combined with visual function training, psychological support, educational resources, life skills training, and behavioral development, can promote long-term benefits in their overall growth and quality of life.

## Author contributions

**Investigation:** Zhangyi Li, Shurui Ke, Ji Sun, Can Li.

**Methodology:** Wenjuan Wan.

**Project administration:** Ke Hu, Can Li.

**Supervision:** Ke Hu, Can Li.

**Writing – original draft:** Zhangyi Li, Shurui Ke, Wenjuan Wan, Ji Sun.

**Writing – review & editing:** Wenjuan Wan, Ke Hu, Can Li.
